# Supplementary material for: Local and global patterns support medical imaging as a biomarker of ageing
Source: Commun Med (Lond). 2026 Jun 13;6:335. doi: 10.1038/s43856-026-01722-3 (PMC13264634; doi:10.1038/s43856-026-01722-3)
Supplement: Supplementary file 2 — Supplementary Material [file 43856_2026_1722_MOESM2_ESM.pdf]

Supplementary Material A Additional Results

A.1 Model Performance

All model performances are visualised in Supplementary Figure A2. The left column shows the performance on the training set, the middle column on the validation set, and the most-right column on the test set. We note that all models tend to overfit on the training set but still generalise well to the validation and the test set. The degree of overfitting is further characterised in Supplementary Figure A1, which shows the residual distributions for the masked and unmasked whole-body age models across training and validation splits. The masked model exhibits closer agreement between splits, supporting its use as a regularisation strategy. The training and validation sets contain subjects without any records of ICD-10 codes and self-reported diseases and are therefore meant to represent healthy ageing patterns. Additionally, the results pre- and post-bias correction, are reported in Table A1 and visualised in Supplementary Figure A3.

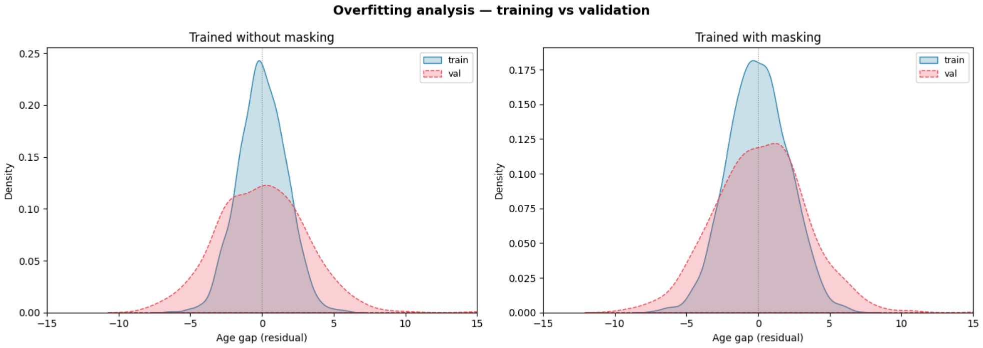

Supplementary Figure A1 Residual distributions for the masked and unmasked whole-body age models
Kernel density estimates of the residual age gap are shown separately for train (blue, solid) and validation (red, dashed) splits, for the unmasked (left) and masked (right) models. A narrower train distribution relative to the validation distribution indicates over-fitting.

Supplementary Table A1 Performance summary of all models before and after bias correction. The results show minimal changes overall, with a general trend of improved performance after correction.

	Before correction			After correction		
**Dataset**	Train MAE	Val MAE	Test MAE	Train MAE	Val MAE	Test MAE
Whole body	1.754 ± 1.12	2.639 ± 1.21	2.719 ± 1.26	1.706 ± 1.31	2.492 ± 1.37	2.564 ± 1.45
Brain	1.657 ± 1.64	2.644 ± 1.34	2.906 ± 1.73	1.500 ± 1.62	2.338 ± 1.35	2.496 ± 1.75
Heart	1.909 ± 4.16	3.572 ± 4.11	3.405 ± 2.20	0.980 ± 1.67	2.634 ± 2.38	3.022 ± 3.34
Spine	0.789 ± 1.39	3.164 ± 1.70	3.279 ± 2.19	0.750 ± 1.32	3.028 ± 1.71	3.164 ± 2.44
Liver	0.882 ± 1.45	4.014 ± 1.83	4.081 ± 1.85	0.841 ± 1.42	3.842 ± 1.87	3.929 ± 1.87
Lungs	0.802 ± 1.16	3.166 ± 1.56	3.255 ± 1.60	0.784 ± 1.15	3.071 ± 1.57	3.152 ± 1.60
Muscle	1.001 ± 1.24	2.702 ± 1.28	2.847 ± 1.34	0.939 ± 1.20	2.627 ± 1.30	2.722 ± 1.36
Intestine	1.056 ± 1.52	3.751 ± 1.92	3.798 ± 2.01	1.033 ± 1.51	3.639 ± 1.93	3.668 ± 2.02

A.2 Chronic Diseases

We summarise the results of our analysis comparing accelerated ageing and specific diseases for all body regions in Figure A4. We note that corresponding visualisations for the whole-body age and the brain age are contained in the main part of this manuscript.

A.3 Lifestyle Features

Figure A5 shows the differences in predicted age versus chronological age for smokers (red) and non-smokers (blue) for different body region ages (heart, liver, spine, muscle, intestine). Smokers tend to show more accelerated ageing compared to non-smokers. Their age prediction lies mostly above the line of perfect prediction (orange), while non-smokers are more evenly distributed showing accelerated and decelerated ageing.

A.4 Survival Analysis

We visualise additional Kaplan Meier curves for the remaining body regions that show the difference in survival probability between accelerated agers ("acc", orange) and decelerated agers ("dec", blue) in Figure A6. The shaded area indicates the confidence interval. For all visualised body regions (lungs, liver, spine, muscle, intestine, heart), 5106 subjects show accelerated ageing and 5234 decelerated ageing.

A.5 Virtual Ageing Model

We here summarise additional results of the Virtual Ageing Model, where we simulate younger body region ages for all regions individually as well as for all accelerated regions and summarise the respective whole-body ages and the corresponding age gaps in Table A2. We highlight that artificially reducing the spine age has the same impact on the whole-body age as simulating decelerated ages for all accelerated body regions. With these experiments, it always needs to be kept in mind that even though a simulated younger region does not impact the overall whole-body age prediction, this can still mean that it is important for the subject to intervene on their body-region ages and, for example, make corresponding lifestyle changes. In this experiment, replacing the spine in the virtual ageing model led to the least accelerated whole-body age with an age gap of +4.06.

Supplementary Table A2 **Impact of regional virtual ageing on whole-body age predictions**

Dataset	Whole-body Age	Age Gap
Spine	63.86	**+4.06**
Heart	67.18	+7.38
Lungs	65.87	+6.07
Liver	65.07	+5.27
Intestine	66.48	+6.68
Muscle	64.97	+5.17
All	64.83	+5.03
Spine, Intestine, Muscle, Lungs	63.99	+4.19

Results of the Virtual Ageing Model for simulating younger ages of local regions in the body. The whole-body age and the age gap indicate the age prediction/age gap of the virtual ageing model after altering the respective region(s). The subject of reference is 59.8 years old and the smallest age gap is achieved by decreasing their spine age (highlighted in **bold**).

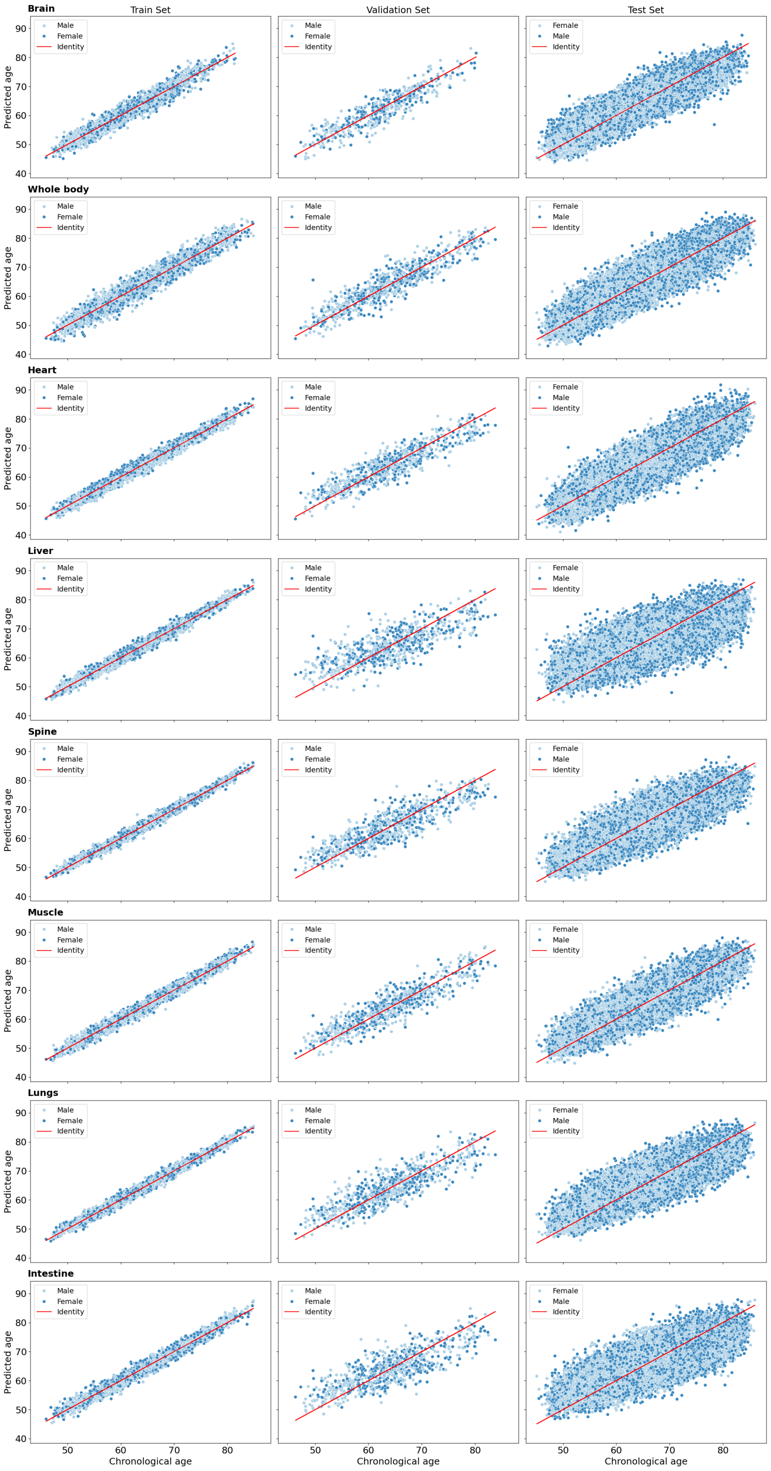

Supplementary Figure A2 Overall model performances

Model performance on the train set (left), the validation set (middle), and the test set (right) for the brain (first row), whole body (second row), heart (third row), liver (fourth row), spine (fifth row), muscle (sixth row), lungs (seventh row), and intestine (eighth row). The red line indicates a perfect prediction and the colours indicate sex (blue: female, dark blue: male).

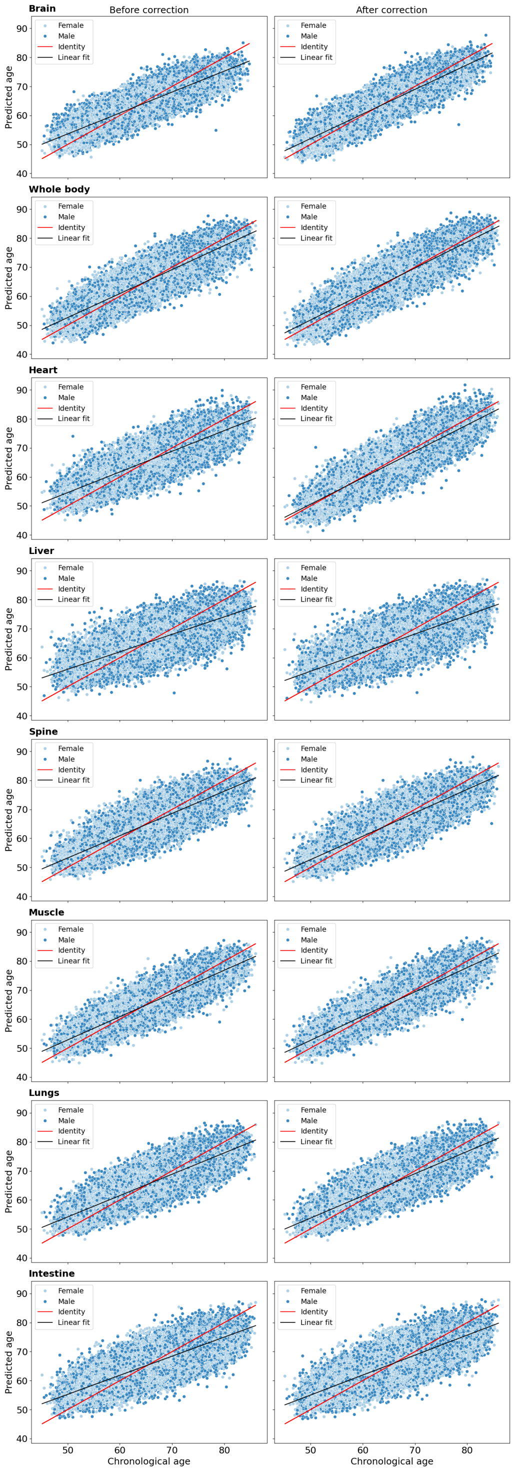

Supplementary Figure A3 Effect of bias correction on model predictions

Predicted age vs. chronological age before bias correction (left) and after bias correction (right) for the brain (first row), whole body (second row), heart (third row), liver (fourth row), spine (fifth row), muscle (sixth row), lungs (seventh row), and intestine (eighth row). The red line indicates a perfect prediction, the black line shows a linear fit to the data, and the colours indicate sex (blue: female, dark blue: male).

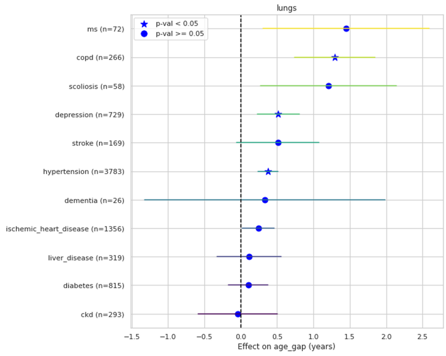

(a) *Lung* age gaps of disease groups

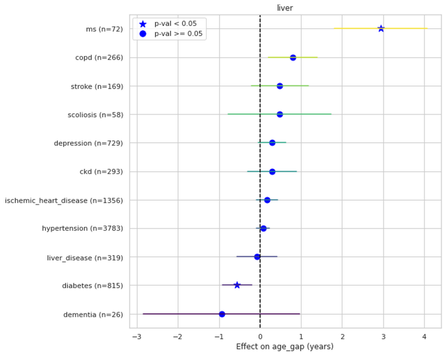

(b) *Liver* age gaps of disease groups

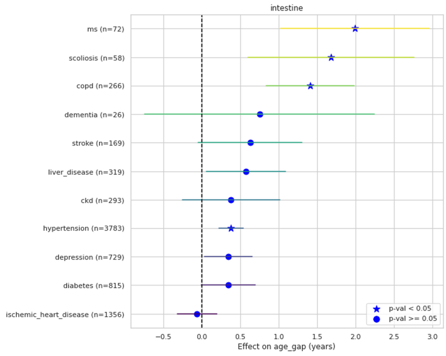

(c) *Intestine* age gaps of disease groups

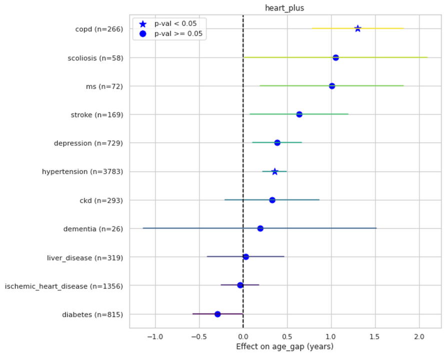

(d) *Heart* age gaps of disease groups

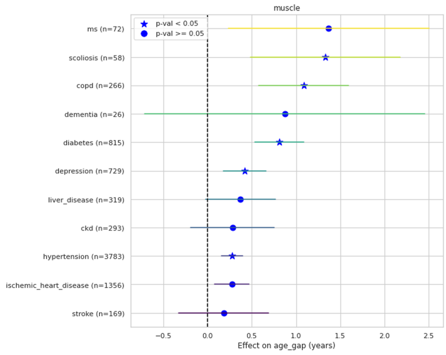

(e) *Muscle* age gaps of disease groups

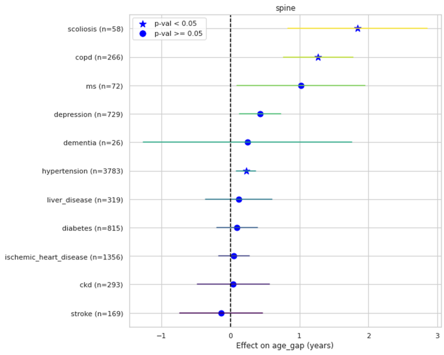

(f) *Spine* age gaps of disease groups

Supplementary Figure A4 Propensity-weighted age gaps across diseases for brain and whole-body datasets Distribution of age gaps for different diseases for (a) the lung, (b) the liver, (c) the intestine, (d) the heart, (e) the muscle, and (f) the spine age predictions. The dotted horizontal (red) line indicates the average age gap of all subjects with diseases (test set). The diseases are ordered by ascending median age gap.

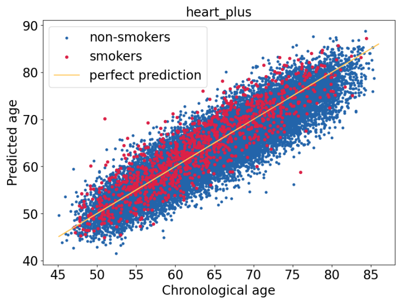

(a) Heart

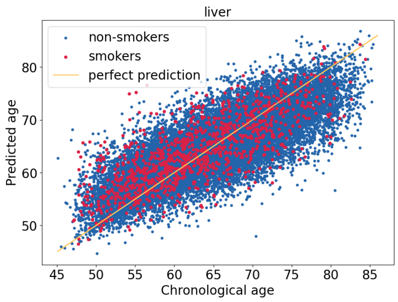

(b) Liver

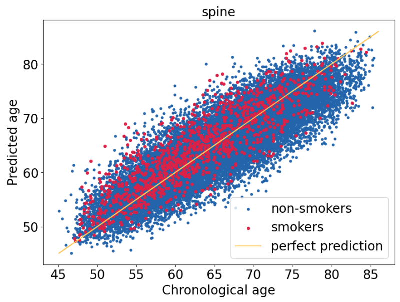

(c) Spine

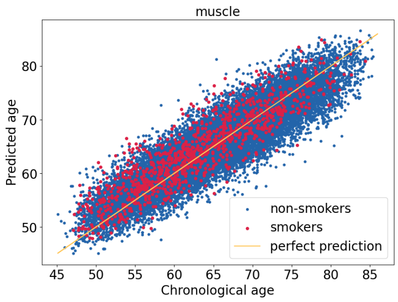

(d) Muscle

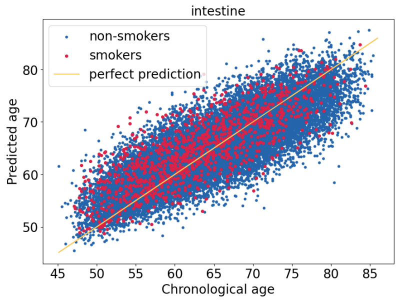

(e) Intestine

Supplementary Figure A5 Body region age predictions by smoking status
Visualisation of the predicted age vs. the chronological age of smokers (red) and non-smokers (blue) for (a) the heart, (b) the liver, (c) the spine, (d) the muscle, and (e) the intestine images. Smokers tend to show more accelerated ageing in all image datasets.

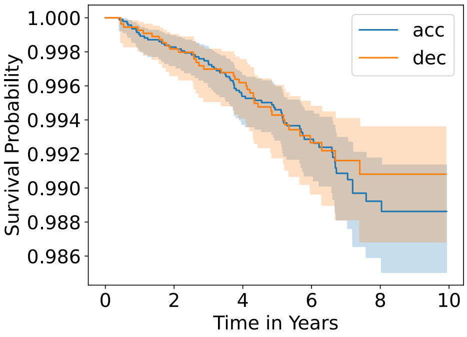

(a) Lungs

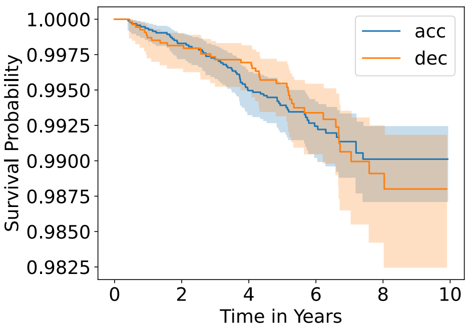

(b) Liver

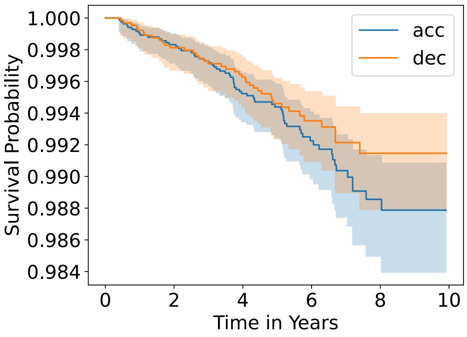

(c) Spine

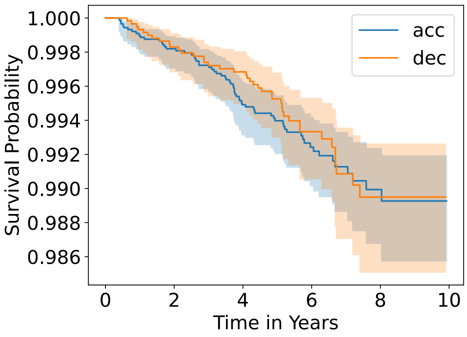

(d) Muscle

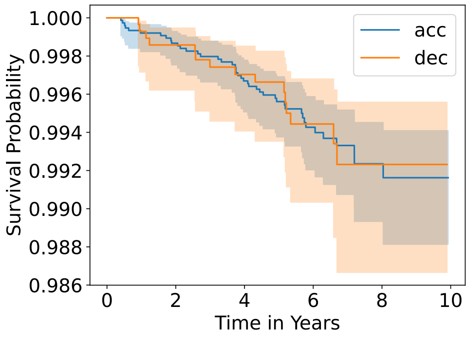

(e) Intestine

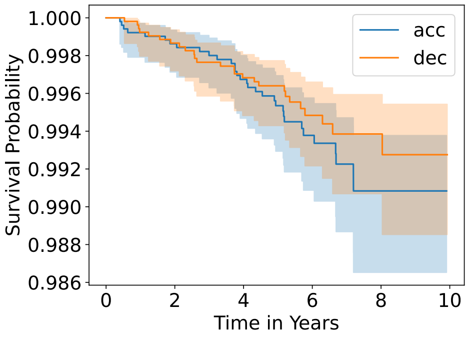

(f) Heart

Supplementary Figure A6 Kaplan Meier curves for body-region ages

This figure compares accelerated (acc, blue) and decelerated (dec, orange) agers for the lungs (a), the liver (b), the spine (c), the muscles (d), the intestine (e) and the heart (f) with the associated error bands.

Supplementary Material B Dataset details

B.1 Summary Dataset

Supplementary Table B3 Baseline characteristics of the whole body dataset

Characteristic	Females (n=26,331)	Males (n=23,335)
Age (years)	64.8 ± 5.2	65.5 ± 5.2
Weight (kg)	69.3 ± 13.4	84.2 ± 13.6
Height (cm)	163.5 ± 6.4	177.2 ± 6.7
Diabetes	2.5%	4.4%
Stroke	0.5%	1.0%
Multiple Sclerosis (MS)	0.4%	0.1%
Dementia	0.1%	0.1%
COPD	1.1%	1.2%
Chronic Kidney Disease (CKD)	1.2%	1.3%
Hypertension	13.0%	19.6%
Ischemic Heart Disease	3.1%	8.5%
Depression	3.8%	2.3%
Scoliosis	0.3%	0.1%
Liver Disease	1.3%	1.6%

Supplementary Table B4 Baseline characteristics of the brain dataset

Characteristic	Females (n=17,350)	Males (n=15,128)
Age (years)	64.7 ± 5.2	65.1 ± 5.2
Weight (kg)	68.9 ± 13.0	83.4 ± 13.3
Height (cm)	163.5 ± 6.4	176.9 ± 6.7
Diabetes	2.4%	3.9%
Stroke	0.5%	0.8%
Multiple Sclerosis (MS)	0.3%	0.1%
Dementia	0.1%	0.1%
COPD	1.1%	1.1%
Chronic Kidney Disease (CKD)	1.1%	1.1%
Hypertension	12.4%	18.2%
Ischemic Heart Disease	3.1%	7.8%
Depression	3.2%	2.0%
Scoliosis	0.3%	0.1%
Liver Disease	1.3%	1.5%

B.2 Dataset overlap

Figure B7 describes the overlap, in terms of subjects, of all datasets (train, val and test).

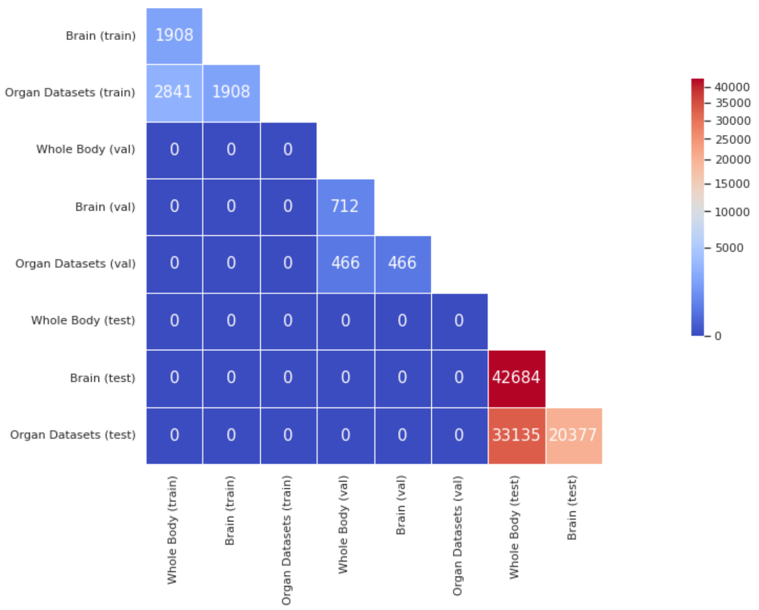

Supplementary Figure B7 Subject overlap of all datasets

Brain subjects are included in the whole-body and organ datasets. Overlap exists within training, validation, and test sets, but not across splits.

B.3 Age gap distribution

Figure B8 describes the predicted age gap distribution by sex for all datasets.

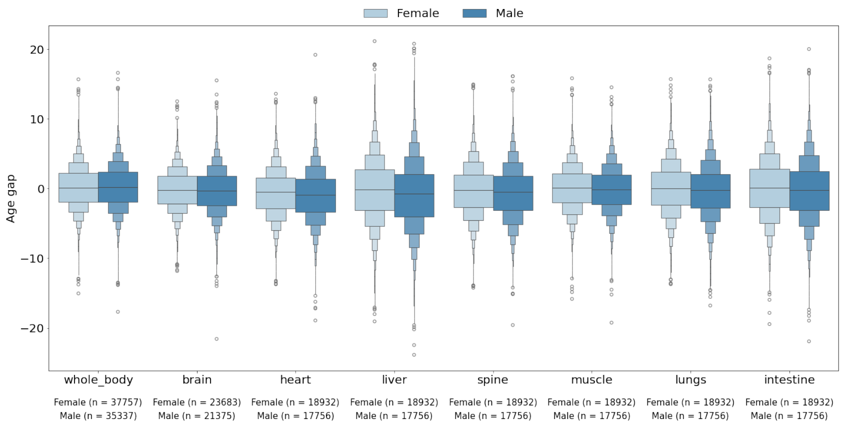

Supplementary Figure B8 Age gap distributions for all body region images for male and female subjects.
