# Supplementary material for: Local and global patterns support medical imaging as a biomarker of ageing
Source: Commun Med (Lond). 2026 Jun 13;6:335. doi: 10.1038/s43856-026-01722-3 (PMC13264634; doi:10.1038/s43856-026-01722-3)
Supplement: Supplementary file 1 — Transparent Peer Review file [file 43856_2026_1722_MOESM1_ESM.pdf]

nature portfolio

Peer Review File

Local and Global Patterns Support Medical Imaging as a Biomarker of Ageing

Corresponding Author: Dr Tamara Mueller

This file contains all reviewer reports in order by version, followed by all author rebuttals in order by version.

Version 0:

Reviewer comments:

Reviewer #1

(Remarks to the Author)
The manuscript presents a large-scale study leveraging UK Biobank MR imaging to investigate biological ageing patterns using deep learning. The authors train separate 3D ResNet-18 models to predict chronological age from various body regions and compute the age gap (predicted minus actual age) as a surrogate biomarker. The study is ambitious in scope, combining predictive modelling, disease association analyses, PheWAS, survival analysis, and a conceptual framework of Digital Twins. While the overall direction is compelling and of potential clinical interest, several methodological aspects require clarification or improvement to ensure scientific rigour and reproducibility.

Major Comments

1. It is unclear whether individuals with multiple scans were considered and, if so, whether proper subject-wise separation was enforced in the train/val/test splits. This is critical to avoid data leakage and overestimated performance. Please clarify whether each subject appears in only one split and how repeated measures were handled (if present).

2. Table 1 mentions "five random repetitions" but also describes a hold-out split with fixed training (80%), validation (20%), and test sets. Furthermore, a validation set is referenced although only healthy subjects are used for training and validation, while unhealthy ones are used exclusively for testing. This creates confusion regarding the actual data splitting strategy and raises questions on whether performance metrics are comparable across organs and conditions. Please revise and clarify the experimental protocol and terminology.

3. In Section 4.2, the authors describe a strategy of randomly masking out parts of the input image during training to force the model to focus on the entire body. While the goal is understandable, this approach is not grounded in established interpretability techniques. There are more formal methods, such as occlusion sensitivity analysis, that would be more appropriate and reproducible. As it stands, this strategy feels arbitrary and lacks rigour.

3. It is not clear whether data augmentation (noise, flips, rotations) was applied globally before data splitting or only within cross-validation folds. To ensure reproducibility and prevent information leakage, augmentation should be applied exclusively within training folds. Please clarify the timing and scope of augmentation in the pipeline.

4. Section 4.2 states that five models were trained for each organ, but there is no detailed description of the model diversity (e.g., different initializations, folds, architectures). Additionally, it is surprising that the same 3D ResNet-18 architecture is applied across all body regions regardless of anatomical or resolution differences. Have you evaluated whether organ-specific architectures might offer performance benefits? This would be important especially for smaller or less informative regions.

5. Section 4.3 introduces a post-hoc linear bias correction method for the predicted age gaps. However, the manuscript does not show the model results before correction. It is good practice to report both pre- and post-bias correction performance

(e.g., MAE, correlation) to assess the real effect of this step and to enable comparison with existing literature.

6. The disease-related analysis in Section 2.3 uses subgroups with widely varying sample sizes (e.g., MS: n=76 vs. hypertension: n=5176), without any discussion of statistical power or potential confounders. Moreover, many conditions considered (e.g., depression, diabetes) often co-occur, especially in ageing cohorts. Have you accounted for comorbidities or confounding factors? A multivariate model or propensity score stratification could improve the robustness of this analysis.

7. The so-called Digital Twin is created by replacing organ images of a subject with those from another subject with similar chronological age but lower predicted biological age. While this demonstrates the effect of organ-level changes, it is more akin to an "ablation" or "image substitution" experiment. A true Digital Twin should involve a generative or physics-informed simulation of plausible ageing trajectories within the same subject, rather than direct substitution from another. Please revise the terminology or justify this framing more thoroughly.

8. The manuscript claims that the source code is available at: https://anonymous.4open.science/r/age_biomarkers-0AE9, but at the time of review the code at this link is inaccessible. Given the methodological complexity and the importance of reproducibility for a study of this scale, access to the full codebase is essential. Please ensure the repository is publicly accessible, complete, and contains detailed documentation and scripts to replicate the results, including model training, data preprocessing, and bias correction. If the link is anonymous for review purposes, a README file or snapshot should be provided through the journal's submission system.

Minor Comments

-The term “healthy” is defined as absence of ICD-10 or self-reported illness codes, which may not fully exclude undiagnosed pathology. This limitation should be acknowledged more explicitly in the Discussion.

-Some sentences are overly vague or speculative (e.g., "we envision this being incorporated into medical workflows..."). Consider toning down these claims or providing more concrete paths to translation.

Reviewer #2

(Remarks to the Author)
This study investigates how medical imaging can serve as biomarkers for biological aging by analyzing 70,000 MRI scans from the UK Biobank using 3D deep learning models. The authors trained ResNet-18 networks to predict chronological age across different body regions (whole body, brain, heart, liver, spine, lungs, muscle, intestine) and calculated "age gaps" between predicted and actual age as measures of accelerated or decelerated aging. The study found significant associations between accelerated aging and diseases (multiple sclerosis showed highest brain age acceleration, scoliosis highest whole-body acceleration), lifestyle factors (smoking strongly accelerated aging, physical activity was protective), and introduced a "Digital Twin" concept for personalized aging pattern analysis. While this paper is well crafted, there are some weaknesses to further improve the quality as follows:

1. The method for adapting pre-trained 2D ImageNet weights to 3D ResNet-18 architecture has not been described in sufficient detail.
2. The reason why the authors chose the model architecture, 3D ResNet-18, is not clear. Would there be any better/other models?
3. The proposed method cannot distinguish between pathological and physiological aging processes. So it is unclear whether observed accelerated aging reflects natural biological variation or underlying disease states.
4. Multiple confounding variables including chronological age, genetic factors, and socioeconomic status are inherently intertwined. Please comment on this.
5. Please comment on the age group as most subjects 60-70 years old with insufficient young/elderly representation.
6. The current study is based on cross sectional design so cannot establish causality or temporal relationships.

Reviewer #3

(Remarks to the Author)

- The first sentence gives a strange impression, revise: Understanding the process of ageing has become a highly desirable goal in human health and disease research.

- The entire abstract, should be revised with a focused presentation on the content of the paper. Furthermore, language polishing should be conducted for the entire paper. At the moment the paper is hard to read.

- In the abstract and introduction, digital twins are mentioned but not discussed. If this is an important topic of the paper, the introduction should introduce and review this concept in sufficient detail. Since Sec 2.6 reports results about this I guess it is

a key topic of the paper, hence, the introduction is clearly lacking this.

- Table 1: Why are no sample details about the six diseases in the bottom part of the table? I suggest to add details.

- Sec 4.1 discussed the data but lacks a discussion of the sample sizes. Are these sufficient? Add a justification.

- Sec 4.8 presents digital twins for imaging but does not explain the underlying concept in detail. (see also introduction). For a general discussion of the concept in a health science context:
https://www.mdpi.com/1422-0067/23/21/13149

- In the discussion section, I am missing a discussion about the connection between images and models because for digital twins one needs a model of the underlying processes. What is the model used in this paper and how is it defined? At the moment I cannot find the digital twin model.

- I am also missing the conclusion section, summarizing briefly all findings.

Reviewer #4

(Remarks to the Author)
Review of "Medical Images as Biomarkers of Ageing – From Global and Local Patterns to Digital Twins"
This study proposes to analyse brain and whole-body scans from the UK Biobank to predict biological age. Conceptually, as the authors note, imaging biomarkers of ageing have been explored before at the single-organ and multi-organ level. Their results are broadly consistent with previous work, showing that organ images can provide a more precise index of biological age and may be useful for predicting age-related morbidity. In line with earlier UK Biobank studies, they also perform a PheWAS analysis.
Major Comments
1. Use of the term "digital twin"
The claim of building a digital twin is not supported by the generally accepted definition of the term. A digital twin should track an individual through time and typically include a mechanistic model. Here, the "digital twin" appears to be a patient specific analysis of a classifier. A more appropriate term should be used to describe this section to avoid confusion.
2. Lack of external validation
The results are not validated in an independent cohort. This is essential to ensure that findings are not overfitted to UK Biobank data. Without such validation, the generalisability of the model is uncertain.
3. Code availability
The code was not made available, Possibly this can change once the paper is accepted.
4. Data clarity
It is unclear how much overlap exists between the brain and whole-body imaging cohorts. This should be explicitly clarified.
5. Feature importance analysis
In the digital twin section, the rationale for not using more conventional feature importance methods—such as SHAP or global sensitivity analysis (GSA)—is not well explained. Employing or justifying these approaches would strengthen the interpretability of the results.
6. Discussion section
The discussion often repeats content from the introduction. It should be streamlined to improve clarity and conciseness.
7. Definition of "validation"
The terminology used for train/validation/test splits may be confusing. In machine learning, "validation" usually refers to an independent test set with known labels. In this paper, "validation" is used to mean confirmation of the authors' hypothesis in

the diseased test set, which is a distinct concept. Clearer terminology is needed. At present, there is no independent validation dataset for the health age network, which is a major limitation.
8. Bias correction
The addition of a bias correction step further underscores the need for an independent healthy validation set.
9. Hypertension and ageing
Could the authors clarify whether hypertension is considered part of “natural ageing”? The removal of all ICD-10 codes would exclude hypertensive cases, which are very common in UK Biobank. This exclusion could bias the training set toward an unexpectedly healthy ageing cohort.
10. Segmentation quality
More detail is needed on the number of faulty segmentations and how they were handled.
Major Concern
The lack of external validation is the most significant limitation of this study. Without it, the robustness and clinical relevance of the findings remain uncertain.

Version 1:

Reviewer comments:

Reviewer #1

(Remarks to the Author)
The authors have substantially addressed all major concerns raised in the previous review.
The revised manuscript is significantly improved in terms of methodological clarity, statistical robustness, and conceptual precision. In particular, the clarification of the data splitting strategy, the introduction of propensity score weighting for disease associations, the inclusion of pre- and post-bias-correction results, and the revision of the “Digital Twin” terminology represent meaningful and appropriate responses.

The study now presents a comprehensive and technically sound investigation of multi-organ ageing patterns from large-scale medical imaging data, with results that are of clear interest to the readership of the journal.

Minor comment

While the renaming of the “Digital Twin” to “Virtual Ageing Model” is appropriate and improves conceptual accuracy, the manuscript would benefit from a slightly more explicit interpretative disclaimer. The current framework relies on cross-subject organ substitution to generate counterfactual scenarios, which should be interpreted as an exploratory intervention analysis rather than a biologically causal or generative simulation of ageing trajectories within an individual. A brief clarification in the Discussion would help prevent overinterpretation by non-technical readers and further strengthen the conceptual positioning of the method.

Reviewer #2

(Remarks to the Author)
While the authors addressed the most of concerns raised by the reviewers, there are lingering concerns as follows:

1. What is the MAE on NAKO before and after linear correction? How do you know a linear correction is sufficient rather than masking poor generalization?
2. If SHAP is not applicable, what alternative interpretability method ensures the Virtual Ageing Model captures biologically meaningful signals rather than artifacts?

Reviewer #4

(Remarks to the Author)
The authors have significantly improved the manuscript, and my major concerns have now been addressed.
Several key methodological choices are currently justified primarily by referencing to community norms or prior usage rather than by evidence within this study. Specifically, the manuscript would be strengthened by providing additional empirical justification for:
• the choice of a single, uniform architecture across all organs,
• the use of the masking strategy during training, and
• the application of the bias correction step.
While these choices are reasonable and commonly used, the current justification largely rests on “others have done this before.” The authors could consider sensitivity analyses, ablations, or supplementary comparisons to strengthen/increase confidence in the results.

Version 2:

Reviewer comments:

Reviewer #2

(Remarks to the Author)
I thank the authors for their careful revision. The authors have addressed prior critiques adequately, and I have no further concerns.

Open Access This Peer Review File is licensed under a Creative Commons Attribution 4.0 International License, which permits use, sharing, adaptation, distribution and reproduction in any medium or format, as long as you give appropriate credit to the original author(s) and the source, provide a link to the Creative Commons license, and indicate if changes were made.
In cases where reviewers are anonymous, credit should be given to 'Anonymous Referee' and the source.
The images or other third party material in this Peer Review File are included in the article's Creative Commons license, unless indicated otherwise in a credit line to the material. If material is not included in the article's Creative Commons license and your intended use is not permitted by statutory regulation or exceeds the permitted use, you will need to obtain permission directly from the copyright holder.
To view a copy of this license, visit https://creativecommons.org/licenses/by/4.0/

Reviewers' comments:

Reviewer #1 (Remarks to the Author):

The manuscript presents a large-scale study leveraging UK Biobank MR imaging to investigate biological ageing patterns using deep learning. The authors train separate 3D ResNet-18 models to predict chronological age from various body regions and compute the age gap (predicted minus actual age) as a surrogate biomarker. The study is ambitious in scope, combining predictive modelling, disease association analyses, PheWAS, survival analysis, and a conceptual framework of Digital Twins. While the overall direction is compelling and of potential clinical interest, several methodological aspects require clarification or improvement to ensure scientific rigour and reproducibility.

Major Comments

1. It is **unclear** whether individuals with multiple scans were considered and, if so, whether proper subject-wise separation was enforced in the train/val/test splits. This is critical to avoid **data leakage** and overestimated performance. Please clarify whether each subject appears in only one split and how repeated measures were handled (if present).

> The UK Biobank includes two imaging visits; however, we use only the first visit in this analysis. As a result, each participant appears only once. We have updated **Section UK Biobank of the Methods** to clarify this:
>
> *“We utilise (a) 73 094 T1-weighted, dual-echo gradient whole-body MRI datasets with a size of 224 × 168 × 363 voxels and a spatial resolution of 2, 23 × 3 × 2, 23mm. The whole-body MR images were acquired in several stations and stitched using the pipeline of [20]. Furthermore, we use (b) 45 058 T1-weighted scull-stripped brain MRI datasets of the UK Biobank with an isotropic spacing of 1mm3 and a size of 160 × 225 × 160 voxels. Although the UK Biobank dataset contains repeat scans for the same participants, we retain only the first one to avoid any data leakage.”*
>
> [20] Lavdas, I. et al. Machine learning in whole-body mri: experiences and challenges from an applied study using multicentre data. Clinical radiology 74, 346–356 (2019).

2. Table 1 mentions "five random repetitions" but also describes a hold-out split with fixed training (80%), validation (20%), and test sets. Furthermore, a validation set is referenced although only healthy subjects are used for training and validation, while unhealthy ones are used exclusively for testing. This creates **confusion regarding the actual data splitting** strategy and raises questions on **whether performance metrics are comparable across organs** and conditions. Please revise and clarify the experimental protocol and terminology.

> Thank you for pointing out this ambiguity. We agree that the original wording could be confusing and have revised the manuscript to clearly describe the experimental protocol and data splits. The term “five random repetitions” refers to five independent training runs with different random seeds, not to repeated re-splitting of the data, following a standard machine learning procedure. The data partitions are fixed; only stochastic training elements vary. Results are reported as the mean and standard deviation across these five runs. Because identical splits and seeds are used across all organs and experiments, performance metrics are directly comparable.
>
> We also clarified the rationale for healthy-only training and validation in the manuscript. As the method follows a healthy-reference paradigm, only healthy UK Biobank (UKB) subjects are used for training and internal validation, with the validation set employed solely for model selection and early stopping (using ML terminology of validation set here). Unhealthy UKB subjects are reserved

exclusively for testing and contains subjects the model has never seen during training. This choice is grounded on the aim to have the model pick up ‘healthy ageing’ only. In addition, we introduce an external validation using the independent NAKO cohort to assess generalisation. The NAKO data are completely unseen during model development, ensuring a clear separation between internal validation, testing on pathology, and external validation. These clarifications are now reflected in Table 1 and the Methods section.

Here is the new extract from the manuscript in **Section UK Biobank of the Methods**:

“We split the dataset into healthy and unhealthy subjects. Healthy subjects are selected as ones that do not have ICD-10 entries or self-reported diseases. “Healthy” subjects are used exclusively for training the model and are divided into a fixed training (80%) and validation (20%) split. The validation set is used solely for model selection and early stopping. “Unhealthy” subjects are held out entirely and used only as a test set to evaluate performance on pathological cases. To assess generalisation, we further introduce an external validation cohort from the NAKO study [21], which is completely unseen during training and model selection. The detailed numbers of samples for all splits and datasets can be found in Table 3. The sample sizes for all training sets are comparable and yield a substantial improvement over the naive mean-age baseline, indicating adequate generalisation. All experiments are repeated five times using different random seeds while keeping the data splits fixed, and results are reported as mean ± standard deviation across runs. We aim for the model to learn to predict the chronological age of healthy subjects and, therefore, be able to identify visual changes in the “unhealthy” subjects of the test set.”

[21] Bamberg, F. et al. Whole-body mr imaging in the german national cohort: rationale, design, and technical background. Radiology 277, 206–220 (2015).

3. In Section 4.2, the authors describe a strategy of randomly masking out parts of the input image during training to force the model to focus on the entire body. While the goal is understandable, this approach is **not grounded in established interpretability techniques**. There are more formal methods, such as **occlusion sensitivity analysis**, that would be more appropriate and reproducible. As it stands, this strategy feels arbitrary and lacks rigour.

Random masking is not employed for interpretability, but rather as a regularisation mechanism designed to enhance model generalisation. Several studies [42, 43] have shown that incorporating such masking improves performance, highlighting the suitability of this method for our purpose. **Section Deep Learning Models of the Methods** has been updated to reflect this and now includes the relevant citations:

“*We use this “masking” as a regularisation technique [28, 29] for the whole-body and the brain age predictors.*”

[28] Zhong, Z., Zheng, L., Kang, G., Li, S. & Yang, Y. Random erasing data augmentation, Vol. 34, 13001–13008 (2020).
[29] DeVries, T. & Taylor, G. W. Improved regularization of convolutional neural networks with cutout. arXiv preprint arXiv:1708.04552 (2017).

3. It is not **clear whether data augmentation** (noise, flips, rotations) was applied globally before data splitting or only within cross-validation folds. To ensure reproducibility and prevent information leakage, augmentation should be applied exclusively within training folds. Please clarify the timing and scope of augmentation in the pipeline.

Data augmentation was applied exclusively to the training set. No augmentation was performed on the validation or test data. Augmentations (noise, flipping, and rotation) were applied online during

training, ensuring no information leakage and full reproducibility. The manuscript has been updated to clarify this point, see **Section Deep Learning Models of the Methods:**

"*Furthermore, we use random data augmentation (addition of noise, rotation) applied exclusively to the training set during training for all models to prevent over-fitting.*"

4. Section 4.2 states that five models were trained for each organ, but there is no detailed description of the model diversity (e.g., different initializations, folds, architectures). Additionally, it is surprising that the same 3D ResNet-18 architecture is applied across all body regions regardless of anatomical or resolution differences. Have you evaluated whether organ-specific architectures might offer performance benefits? This would be important especially for smaller or less informative regions.

We thank the reviewer for their comment. The reason for using 3D ResNet-18 is because it is a well-established method in similar works [15]. Because it has been shown to provide competitive performance, while balancing computational cost, memory requirements, and robustness [40]. Furthermore, using the same architecture highly increases flexibility and versatility of our proposed approach. This can be applied to other organs, image types, or regions of interest as well, without the need for further cost-intensive model selection. We recognise that optimising the architecture per method and choosing the best performing one might have some value but due to computational limitations we refrain and opt for the same architecture. We have updated the **Section Deep Learning Models of the Methods** to include this rationale:

"*We use 3D ResNet18 for all models due to their established use in related work [15, 38] and their favourable balance between performance and computational efficiency. A consistent architecture also improves the flexibility and generalisability of our approach without requiring additional model selection.*"

[15] Bashyam VM, Erus G, Doshi J, Habes M, Nasrallah IM, Truelove-Hill M, et al. MRI signatures of brain age and disease over the lifespan based on a deep brain network and 14 468 individuals worldwide. Brain. 2020;143:2312–24.
[23] Singh, S. P., Wang, L., Gupta, S., Goli, H., Padmanabhan, P., & Gulyás, B. (2020). 3D Deep Learning on Medical Images: A Review. Sensors, 20(18), 5097. https://doi.org/10.3390/s20185097

5. Section 4.3 introduces a post-hoc linear bias correction method for the predicted age gaps. However, the manuscript does not show the model results before correction. It is good practice to report both pre- and post-bias correction performance (e.g., MAE, correlation) to assess the real effect of this step and to enable comparison with existing literature.

Following the reviewer's recommendation, we have added **Supplementary Table A1** to the Appendix, which summarises the pre- and post-bias-correction performance. The results show minimal changes overall, with a general trend of improved performance after correction.

6. The disease-related analysis in Section 2.3 uses subgroups with widely varying sample sizes (e.g., MS: n=76 vs. hypertension: n=5176), **without any discussion of statistical power or potential confounders**. Moreover, many conditions considered (e.g., depression, diabetes) often co-occur, especially in ageing cohorts. **Have you accounted for comorbidities or confounding factors? A multivariate model or propensity score stratification could improve the robustness of this analysis.**

We thank the reviewer for this suggestion. We have revised the disease-related analysis **Sections Chronic Diseases of the Methods and Results** and updated Figure 3 (now Figure 4) to address the concerns raised. Specifically, we now apply propensity score weighting to account for

differences in sample size and potential confounding effects. The model adjusts for age, sex, height, weight and smoking status. The updated results are highly consistent with the original findings (figure below), but now additionally provide confidence intervals and statistical significance estimates, thereby strengthening the robustness and interpretability of the analysis. We note that, because not all covariates are available for every subject, the sample size varies slightly from the previous analysis but minimally affects the estimated age gaps. **Section Chronic Diseases of the Methods** now describes to protocol:

“We use the UK Biobank fields 41270 for the ICD-10 records and 20002 for the self-reported non-cancer illness codes to determine, whether a subject is counted to be in a specific disease sub-group. We furthermore use the UK Biobank field 41280 to determine the date of diagnosis of the ICD-10 code. We only select subjects that have a record of the specific disease before the time of the imaging assessment or within one year after. Our goal is to evaluate ageing profiles for different diseases, not predicting disease development. Therefore, we are primarily interested in subjects that already show signs of named diseases in their MR images. To account for sample size differences and potential confounding effects, we perform a propensity score weighting [36] on the age gaps for each disease. Propensity scores were estimated separately for each disease using logistic regression with age, sex, weight, height and smoking status (UK Biobank field 20116) as covariates. They were then used to compute stabilised inverse probability weights, which were truncated at the 1st and 99th percentiles to limit the influence of extreme weights. Weighted least squares regression models were then fitted with the biological age gap as the outcome and disease status as the predictor. Effect estimates represent adjusted mean differences in biological age gap between diseased and non-diseased participants. We report adjusted coefficients, 95% CIs and Bonferroni-corrected P-values for the 11 disease groups.“

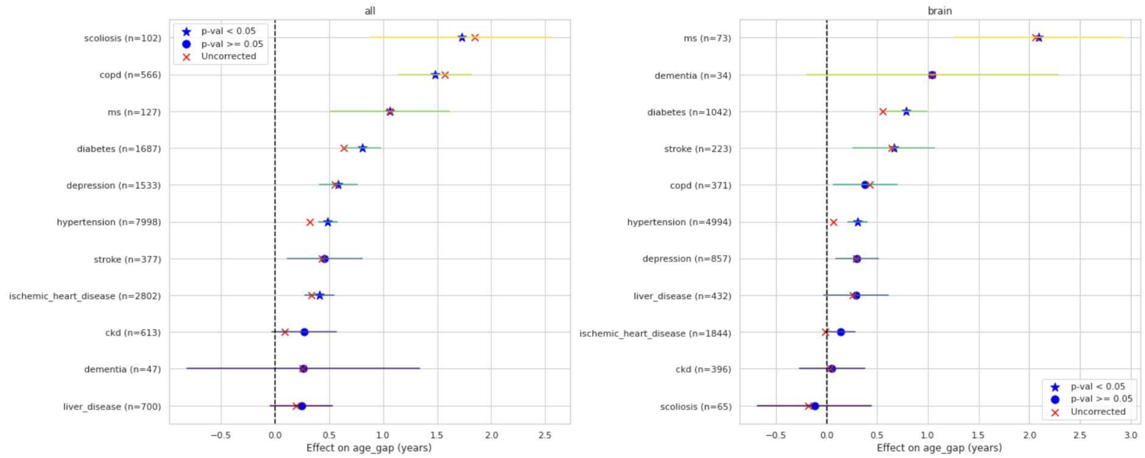

[36] Austin, P. C. An introduction to propensity score methods for reducing the effects of confounding in observational studies. Multivariate behavioral research 46, 399–424 (2011).

7. The so-called Digital Twin is created by replacing organ images of a subject with those from another subject with similar chronological age but lower predicted biological age. While this demonstrates the effect of organ-level changes, **it is more akin to an "ablation" or "image substitution" experiment.** A true Digital Twin should involve a generative or physics-informed simulation of plausible ageing trajectories within the same subject, rather than direct substitution from another. **Please revise the terminology or justify this framing more thoroughly.**

In the original version of the manuscript, we referred to this procedure as a “Digital Twin”. Following the reviewer’s comment, we agree that the term *Digital Twin* is typically reserved for generative or physics-informed simulations that reproduce plausible temporal trajectories within the same individual. Our method instead relies on organ-level image substitution across subjects to explore

counterfactual scenarios. To more accurately reflect the methodological scope and avoid the stronger implications of a full Digital Twin, we now use the term **Virtual Ageing Model**, and we have revised the entire manuscript accordingly.

8. The manuscript claims that the source code is available at: https://anonymous.4open.science/r/age_biomarkers-0AE9, but at the time of review the code at this link is inaccessible. Given the methodological complexity and the importance of reproducibility for a study of this scale, access to the full codebase is essential. Please ensure the repository is publicly accessible, complete, and contains detailed documentation and scripts to replicate the results, including model training, data preprocessing, and bias correction. If the link is anonymous for review purposes, a README file or snapshot should be provided through the journal's submission system.

We thank the reviewer for bringing this to our attention. The link to the codebase has been restored.

Minor Comments

-The term “healthy” is defined as absence of ICD-10 or self-reported illness codes, which may not fully exclude undiagnosed pathology. **This limitation should be acknowledged** more explicitly in the Discussion.

We agree with the reviewer and have included this limitation in **Section 4 (Discussion and Conclusion)** of our manuscript:

“We also highlight the complexity of the ageing process, with pathological conditions superimposed and resulting from a multitude of impact factors that can change over time. These potential confounding variables are not taken into account in this analysis. In future modelling approaches, other components of the ageing process, such as genetics, the immune system, or behavioural components should be taken into account, to frame a more diverse and robust ageing profile for individuals. Additionally, pathological and physiological ageing phenotypes cannot be distinguished with this method. The age predictors are designed on a “healthy” cohort to model normative ageing patterns, but deviations from this baseline may arise from natural biological variation but also subclinical conditions or undiagnosed diseases. Therefore, an accelerated age should be interpreted as indicating a divergence from healthy ageing trajectories, not as a diagnostic marker or a causal indicator of a disease, which would require future work, including long-term follow-up data and targeted studies. We define our “healthy” cohort as subjects that do not have an ICD-10 record and no self-reported diseases, which might not be a sufficient characteristic to ensure their ageing is “healthy”, as some healthy subjects may harbour undetected conditions. On the contrary, this strict criterion excludes subjects with hypertension, a highly prevalent but highly treatable condition in older subjects, which might not greatly interfere with a healthy ageing process. Despite its limitations, this is a commonly used approach in current studies [12]. Finally, because our study is cross-sectional, it cannot establish causality or model temporal ageing trajectories, highlighting the need for longitudinal imaging to investigate how ageing patterns evolve within individuals.”

[12] Tian, Y. E. et al. Heterogeneous aging across multiple organ systems and prediction of chronic disease and mortality. Nature medicine 29, 1221–1231 (2023).

-Some sentences are overly vague or speculative (e.g., "we envision this being incorporated into medical workflows..."). Consider toning down these claims or providing more concrete paths to translation.

We have, to the best of our capacity, limited the use of speculative or vague phrasing.

For example we have changed this phrasing:
“Finally, by creating a Digital Twin of a subject, we can detect their most accelerated body regions, which can guide recommendations for interventions and lifestyle changes. We envision this to be incorporated into medical workflows and a step towards more personalised medicine.”
into:
“Finally, by creating a Digital Twin of a subject, we can detect their most accelerated body regions, which can guide recommendations for interventions and lifestyle changes and potentially be incorporated into clinical workflows, as a step towards more personalised medicine.”

And this:

“Circling back to the defined characteristics of [8] that ageing biomarkers should meet, we can conclude that medical images can be used as biomarkers for ageing but they are no perfect biological age indicators.”
into:
“Circling back to the defined characteristics of [8] that ageing biomarkers should meet, we can conclude that medical images are good biomarker candidates for ageing but they are no perfect biological age indicators.”

[8] Butler, R. N. et al. Aging: the reality: biomarkers of aging: from primitive organisms to humans. The Journals of Gerontology Series A: Biological Sciences and Medical Sciences 59, B560–B567 (2004).

Reviewer #2 (Remarks to the Author):

This study investigates how medical imaging can serve as biomarkers for biological aging by analyzing 70,000 MRI scans from the UK Biobank using 3D deep learning models. The authors trained ResNet-18 networks to predict chronological age across different body regions (whole body, brain, heart, liver, spine, lungs, muscle, intestine) and calculated "age gaps" between predicted and actual age as measures of accelerated or decelerated aging. The study found significant associations between accelerated aging and diseases (multiple sclerosis showed highest brain age acceleration, scoliosis highest whole-body acceleration), lifestyle factors (smoking strongly accelerated aging, physical activity was protective), and introduced a "Digital Twin" concept for personalized aging pattern analysis. While this paper is well crafted, there are some weaknesses to further improve the quality as follows:

1. The method for adapting pre-trained 2D ImageNet weights to 3D ResNet-18 architecture has not been described in sufficient detail.

Pre-trained 2D ImageNet weights are transferred to the 3D ResNet-18 by linear inflation along the depth dimension, i.e., 2D convolutional kernels are replicated across the third dimension and appropriately scaled. This is a common and well-established practice for initialising 3D CNNs from 2D pre-trained models and has been widely used in prior work [40]. We have clarified the weight adaptation procedure in **Section (Deep Learning Models of the Methods**:

"For all models, we use the pre-trained 2D weights from ImageNet that are provided by PyTorch [24] and transfer them to the 3D setting by linear inflation along the depth dimension [25]."

[24] Paszke, A. et al. Pytorch: An imperative style, high-performance deep learning library. CoRR abs/1912.01703 (2019). URL http://arxiv.org/abs/1912.01703
[25] Carreira, Joao, and Andrew Zisserman. "Quo vadis, action recognition? a new model and the kinetics dataset." proceedings of the IEEE Conference on Computer Vision and Pattern Recognition. 2017.

2. The reason why the authors chose the model architecture, 3D ResNet-18, is not clear. Would there be any better/other models?

The reason for using 3D ResNet-18 is because it is a well-established method in similar works [15]. Because it has been shown to provide competitive performance, while balancing computational cost, memory requirements, and robustness [38]. We have added the relevant references to **Section Deep Learning Models of the Methods** of the manuscript:

"*We use 3D ResNet18 for all models due to their established use in related work [15, 23] and their favourable balance between performance and computational efficiency. A consistent architecture also improves the flexibility and generalisability of our approach without requiring additional model selection.*"

[15] Bashyam VM, Erus G, Doshi J, Habes M, Nasrallah IM, Truelove-Hill M, et al. MRI signatures of brain age and disease over the lifespan based on a deep brain network and 14 468 individuals worldwide. Brain. 2020;143:2312–24.
[23] Singh, S. P., Wang, L., Gupta, S., Goli, H., Padmanabhan, P., & Gulyás, B. (2020). 3D Deep Learning on Medical Images: A Review. Sensors, 20(18), 5097. https://doi.org/10.3390/s20185097

3. The proposed method cannot distinguish between pathological and physiological aging processes. So it is unclear whether observed accelerated aging reflects natural biological variation or underlying disease states.

We agree that our method does not explicitly disentangle physiological from pathological ageing. The age predictors are trained on a "healthy" subset to model normative ageing patterns, but deviations from this baseline may arise from natural biological variation, subclinical conditions, or undiagnosed disease. Our accelerated ageing signal should therefore be interpreted as indicating a divergence from healthy ageing trajectories, not as a diagnostic marker or a causal indicator of disease. We have clarified this limitation in **Section 4 (Discussion and Conclusion)** of our manuscript:

"We also highlight the complexity of the ageing process, with pathological conditions superimposed and resulting from a multitude of impact factors that can change over time. These potential confounding variables are not taken into account in this analysis. In future modelling approaches, other components of the ageing process, such as genetics, the immune system, or behavioural components should be taken into account, to frame a more diverse and robust ageing profile for individuals. Additionally, pathological and physiological ageing phenotypes cannot be distinguished with this method. The age predictors are designed on a "healthy" cohort to model normative ageing patterns, but deviations from this baseline may arise from natural biological variation but also subclinical conditions or undiagnosed diseases. Therefore, an accelerated age should be interpreted as indicating a divergence from healthy ageing trajectories, not as a diagnostic marker or a causal indicator of a disease, which would require future work, including long-term follow-up data and targeted studies. We define our "healthy" cohort as subjects that do not have an ICD-10 record and no self-reported diseases, which might not be a sufficient characteristic to ensure their ageing is "healthy", as some healthy subjects may harbour undetected conditions. On the contrary, this strict criterion excludes subjects with hypertension, a highly prevalent but highly treatable condition in older subjects, which might not greatly interfere with a healthy ageing process. Despite its limitations, this is a commonly used approach in current studies [12]. Finally, because our study is cross-sectional, it cannot establish causality or model temporal ageing trajectories, highlighting the need for longitudinal imaging to investigate how ageing patterns evolve within individuals"

[12] Tian, Y. E. et al. Heterogeneous aging across multiple organ systems and prediction of chronic disease and mortality. Nature medicine 29, 1221–1231 (2023).

4. Multiple confounding variables including chronological age, genetic factors, and socioeconomic status are inherently intertwined. Please comment on this.

We agree with the reviewer that these elements could impact the analysis greatly. We have revised the disease-related analysis **Sections Chronic Diseases of the Methods and Results** and updated Figure 3 (now Figure 4) to address the concerns raised. Specifically, we now apply propensity score weighting to account for differences in sample size and potential confounding. The model adjusts for age, sex, height, weight and smoking status. The updated results are highly consistent with the original findings (figure below), but now additionally provide confidence intervals and statistical significance estimates, thereby strengthening the robustness and interpretability of the analysis. We note that, because not all covariates are available for every subject, the sample size varies slightly from the previous analysis but minimally affects the estimated age gaps. Section 4.5 now describes to protocol:

"We use the UK Biobank fields 41270 for the ICD-10 records and 20002 for the self-reported non-cancer illness codes to determine, whether a subject is counted to be in a specific disease sub-group. We furthermore use the UK Biobank field 41280 to determine the date of diagnosis of the ICD-10 code. We only select subjects that have a record of the specific disease before the time

of the imaging assessment or within one year after. Our goal is to evaluate ageing profiles for different diseases, not predicting disease development. Therefore, we are primarily interested in subjects that already show signs of named diseases in their MR images. To account for sample size differences and potential confounding effects, we perform a propensity score weighting [36] on the age gaps for each disease. Propensity scores were estimated separately for each disease using logistic regression with age, sex, weight, height and smoking status (UK Biobank field 20116) as covariates. They were then used to compute stabilised inverse probability weights, which were truncated at the 1st and 99th percentiles to limit the influence of extreme weights. Weighted least squares regressio models were then fitted with the biological age gap as the outcome and disease status as the predictor. Effect estimates represent adjusted mean differences in biological age gap between diseased and non-diseased participants. We report adjusted coefficients, 95% CIs and Bonferroni-corrected P-values for the 11 disease groups.“

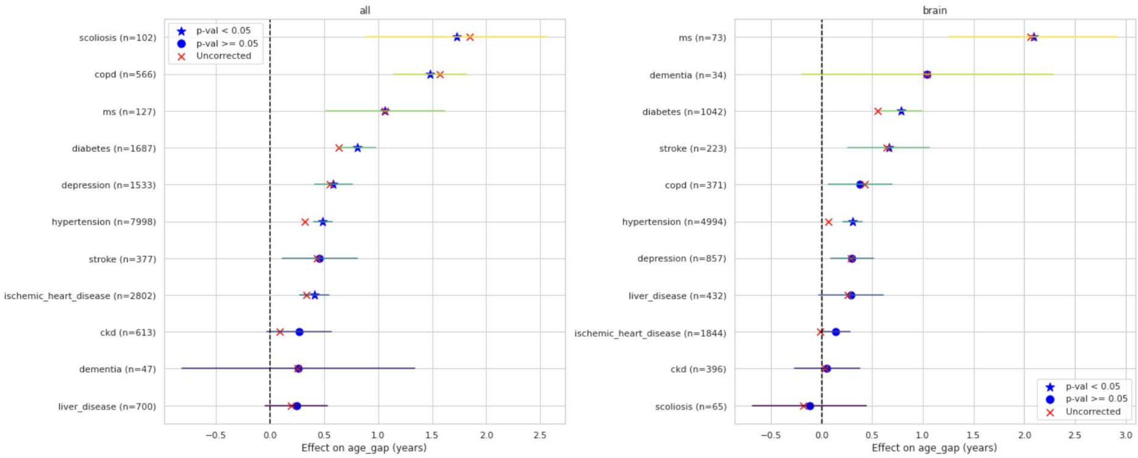

However, confounding variables may still have potential effects, we have discussed this in **Section 4 (Discussion and Conclusion)** as following:

“*We also highlight the complexity of the ageing process, with pathological conditions superimposed and resulting from a multitude of impact factors that can change over time. These potential confounding variables are not taken into account in this analysis. In future modelling approaches, other components of the ageing process, such as genetics, the immune system, or behavioural components should be taken into account, to frame a more diverse and robust ageing profile for individuals.”*

[36] Austin, P. C. An introduction to propensity score methods for reducing the effects of confounding in observational studies. Multivariate behavioral research 46, 399–424 (2011).

5. Please comment on the age group as most subjects 60-70 years old with insufficient young/elderly representation.

We recognise this as a limitation and have highlighted the future work of using a dataset with a larger age range in **Section 4 (Discussion and Conclusion)**:

“While our work renders interesting results about global and local ageing patterns in medical images, we note some limitations. Firstly, the dataset has a limited age range with most subjects centered around 60-70 years old, which makes age prediction especially challenging for under-respresented age groups. This is a very common issue in such ageing datasets that most works address by adding a bias correction step to the pipeline as an effort to mitigate the effects of

such a narrow and unbalanced age range [52]. However, an investigation of a dataset with a wider age range would be highly interesting.“

[52] Azzam, M. et al. A review of artificial intelligence-based brain age estimation and its applications for related diseases. Briefings in Functional Genomics 24, elae042 (2025).

6. The current study is based on cross sectional design so cannot establish causality or temporal relationships.

We agree that the cross-sectional nature of the study prevents causal inference or assessment of temporal ageing dynamics. We now explicitly state this limitation in **Section 4 (Discussion and Conclusion)** and highlight longitudinal imaging as an important direction for future work:

“We also highlight the complexity of the ageing process, with pathological conditions superimposed and resulting from a multitude of impact factors that can change over time. These potential confounding variables are not taken into account in this analysis. In future modelling approaches, other components of the ageing process, such as genetics, the immune system, or behavioural components should be taken into account, to frame a more diverse and robust ageing profile for individuals. Additionally, pathological and physiological ageing phenotypes cannot be distinguished with this method. The age predictors are designed on a “healthy” cohort to model normative ageing patterns, but deviations from this baseline may arise from natural biological variation but also subclinical conditions or undiagnosed diseases. Therefore, an accelerated age should be interpreted as indicating a divergence from healthy ageing trajectories, not as a diagnostic marker or a causal indicator of a disease, which would require future work, including long-term follow-up data and targeted studies. We define our “healthy” cohort as subjects that do not have an ICD-10 record and no self-reported diseases, which might not be a sufficient characteristic to ensure their ageing is “healthy”, as some healthy subjects may harbour undetected conditions. On the contrary, this strict criterion excludes subjects with hypertension, a highly prevalent but highly treatable condition in older subjects, which might not greatly interfere with a healthy ageing process. Despite its limitations, this is a commonly used approach in current studies [12]. Finally, because our study is cross-sectional, it cannot establish causality or model temporal ageing trajectories, highlighting the need for longitudinal imaging to investigate how ageing patterns evolve within individuals”

[12] Tian, Y. E. et al. Heterogeneous aging across multiple organ systems and prediction of chronic disease and mortality. Nature medicine 29, 1221–1231 (2023).

Reviewer #3 (Remarks to the Author):

- The first sentence gives a strange impression, revise: Understanding the process of ageing has become a highly desirable goal in human health and disease research.

- The **entire abstract, should be revised** with a focused presentation on the content of the paper. Furthermore, language polishing should be conducted for the entire paper. **At the moment the paper is hard to read**.

> The abstract has been rewritten entirely to better describe the content of the paper, including the first sentence, which has been revised. We have, furthermore, to the best of our capacity, revised the entire manuscript for improved clarity. This is new abstract of our manuscript:
>
> "**Background**: Understanding human ageing across multiple organs is essential for characterising individual health trajectories and identifying abnormal ageing processes. Multi-organ imaging provides an opportunity to quantify biological ageing beyond chronological age. The aim of this study is to assess organ-specific and whole-body ageing patterns and their associations with disease and lifestyle factors.
> **Methods**: In this large-scale study, we evaluate biological ageing patterns using 70,000 MRI scans from the UK Biobank and the German National Cohort. We employ 3D ResNet-18 models to predict chronological age from various body regions (brain, heart, liver, spine, lungs, muscle, and intestine) and the whole body. From these predictions, we derive "age gaps" relative to a strictly healthy reference cohort, which enables the identification of accelerated ageing patterns. We then evaluate associations with chronic diseases and lifestyle factors, and a virtual ageing framework was developed to explore counterfactual scenarios by substituting anatomical regions across subjects, quantifying local impacts on global biological age.
> **Results**: Here we show significant associations between detected accelerated ageing and specific chronic diseases, including multiple sclerosis and chronic obstructive pulmonary disease, as well as lifestyle factors such as smoking and physical activity. Virtual substitution of anatomical regions demonstrates that local substitutions can influence global ageing patterns.
> **Conclusions**: This study demonstrates that multi-organ imaging enables the detection of abnormal ageing patterns at both local and global levels. The presented framework provides a foundation for improved risk stratification and supports the development of personalised approaches to health assessment and disease prevention."

- In the abstract and introduction, **digital twins are mentioned but not discussed**. If this is an important topic of the paper, the introduction should introduce and review this concept in sufficient detail. Since Sec 2.6 reports results about this I guess it is a key topic of the paper, hence, the introduction is clearly lacking this.

> We thank the reviewer for this comment. We agree that the concept of a digital twin was not sufficiently introduced or discussed in the original version of the manuscript. To avoid confusion and overstatement, we have therefore removed the term "Digital Twin" entirely from the manuscript. Instead, we now refer to this framework as a Virtual Ageing Model, which more accurately reflects the scope and intent of our work.

- Table 1: Why are no sample details about the six diseases in the bottom part of the table? I suggest to add details.

> With the bracket notation, we denote that the six organ datasets are derived from the same cohort and therefore have the same sample sizes. We have clarified this further in the table caption.

- Sec 4.1 discussed the data but lacks a discussion of the sample sizes. Are these sufficient? Add a justification.

We have added a discussion in **Section UK Biobank of the Methods** about the sample size:

"The sample sizes for all training sets are comparable and yield a substantial improvement over the naive mean-age baseline, indicating adequate generalisation."

- Sec 4.8 presents digital twins for imaging but **does not explain the underlying concept in detail**. (see also introduction). For a general discussion of the concept in a health science context: https://www.mdpi.com/1422-0067/23/21/13149

See below.

- In the discussion section, I am **missing a discussion about the connection between images and models** because for digital twins one needs a model of the underlying processes. What is the model used in this paper and how is it defined? At the moment I cannot find the digital twin model.

In the original version of the manuscript, we referred to the procedure of replacing an organ with another to investigate increased/decreased age gap as a "Digital Twin." Following the reviewer's comment, we agree that the term *Digital Twin* is typically reserved for generative or physics-informed simulations that reproduce plausible temporal trajectories within the same individual. Our method instead relies on organ-level image substitution across subjects to explore counterfactual scenarios. To more accurately reflect the methodological scope and avoid the stronger implications of a full Digital Twin, we now use the term Virtual Ageing Model, and we have revised the manuscript accordingly.

- I am also missing the conclusion section, summarizing briefly all findings.

We have renamed the 'Discussion' section into 'Discussion and Conclusion' and have added a more detailed conclusion summary of our findings:

"We show that medical images combined with ML methods can be valid biomarker candidates for ageing. With the full 3D volume of MR images, we achieve accurate age gap predictions and therefore reliable results regarding accelerated and decelerated ageing. Using images eliminates the necessity of selecting features and relying on e.g. self-reported features, both prone to introduce human bias. Morevoer, our approach is highly general and can be applied to arbitrary input data, including higher resolution or functional imaging data. We show that brain age is less correlated to the ageing of the whole body and the selected local regions, indicating a more detached ageing process between body and brain. Finally, by creating a Virtual Ageing Model for a subject, we can detect their most accelerated body regions, which can guide recommendations for interventions and lifestyle changes and potentially be incorporated into clinical workflows, as a step towards more personalised medicine."

Reviewer #4 (Remarks to the Author):

Review of “Medical Images as Biomarkers of Ageing – From Global and Local Patterns to Digital Twins”
This study proposes to analyse brain and whole-body scans from the UK Biobank to predict biological age. Conceptually, as the authors note, imaging biomarkers of ageing have been explored before at the single-organ and multi-organ level. Their results are broadly consistent with previous work, showing that organ images can provide a more precise index of biological age and may be useful for predicting age-related morbidity. In line with earlier UK Biobank studies, they also perform a PheWAS analysis.

Major Comments
1. Use of the term “digital twin”
The claim of building a digital twin is not supported by the generally accepted definition of the term. A digital twin should track an individual through time and typically include a mechanistic model. Here, the “digital twin” appears to be a patient specific analysis of a classifier. A more appropriate term should be used to describe this section to avoid confusion.

In the original version of the manuscript, we referred to this procedure as a “Digital Twin.” Following the reviewer’s comment, we agree that the term *Digital Twin* is typically reserved for generative or physics-informed simulations that reproduce plausible temporal trajectories within the same individual. Our method instead relies on organ-level image substitution across subjects to explore counterfactual scenarios. To more accurately reflect the methodological scope and avoid the stronger implications of a full Digital Twin, we now use the term Virtual Ageing Model, and we have revised the manuscript accordingly.

2. Lack of external validation
The results are not validated in an independent cohort. This is essential to ensure that findings are not overfitted to UK Biobank data. Without such validation, the generalisability of the model is uncertain.

We thank the reviewer for this suggestion and have consequently added the NAKO data as an external validation cohort. We have used the previous models (pretrained on the UK Biobank) to predict ages on the NAKO data with a linear correction step to account for the domain shift. We note that the use of a linear correction is motivated by the assumption that the rate of biological ageing captured by the model is maintained across cohorts, while cohort-specific effects such as scanner type or acquisition protocols primarily induce a global offset. Under this assumption, a linear correction of the intercept is sufficient to account for domain shift without retraining. We then reproduced the smoking analysis and observed similar behaviours to those in the UK Biobank, further supporting the biological validity and generalisability of the learned ageing signal.

We have introduced **Sections External Validation to the Methods and Results** to reflect this:

“*To assess the generalisability of the models and analyses and assess the reproducibility of our findings we introduce an external validation on the German National Cohort (NAKO) [21]. The same models were applied on this external validation set with a linear correction step (more details can be found in section 4.7. Figure 6 displays the predicted ages for smokers and non-smokers for the whole body and the lungs on NAKO. The mean whole-body age gap of smokers is +0.730 years (n = 1271), while the mean age gap of non-smokers is -0.941 years (n = 6077). The mean lung age gap of smokers +0.727 (n=1177), and the mean age gap of non-smokers is -0.310 (n = 5571), supporting findings on the UK Biobank (Section 2.4)*”

[21] Bamberg, F. et al. Whole-body mr imaging in the german national cohort: rationale, design, and technical background. Radiology 277, 206–220 (2015)

3. Code availability
The code was not made available, Possibly this can change once the paper is accepted.

The link to the codebase has been restored.

4. Data clarity
It is unclear how much overlap exists between the brain and whole-body imaging cohorts. This should be explicitly clarified.

A figure with the overlap between all cohorts has been added to the **Supplementary Material B2**.

5. Feature importance analysis
In the digital twin section, the rationale for not using more conventional feature importance methods—such as SHAP or global sensitivity analysis (GSA)—**is not well explained**. Employing or justifying these approaches would strengthen the interpretability of the results.

Our goal in the Digital Twin experiments was to quantify inter-organ dependencies at the level of predicted regional ages rather than to interpret voxel-level features of the deep learning models. Conventional methods such as SHAP or GSA are well-suited to feature-level interpretability for tabular input or for explaining a model's internal representations, but they are less directly applicable to our setting.

6. Discussion section
The **discussion often repeats content from the introduction**. It should be streamlined to improve clarity and conciseness.

We have adapted large parts of the discussion section, minimising the overlap between the Discussion and the Introduction.

7. Definition of "validation"
The **terminology used for train/validation/test splits may be confusing**. In machine learning, "validation" usually refers to an independent test set with known labels. In this paper, "validation" is used to mean confirmation of the authors' hypothesis in the diseased test set, which is a distinct concept. Clearer terminology is needed. At present, there is no independent validation dataset for the health age network, which is a major limitation.

We thank the reviewer for this comment and have clarified the terminology. The validation se**t** consists of held-out healthy UK Biobank subjects used only for model selection and early stopping, not for testing or hypothesis confirmation. The test set contains only unhealthy subjects to evaluate deviations from healthy ageing.
To provide an independent evaluation, we added an external validation cohort from the NAKO dataset, which is entirely separate from UK Biobank and was not used during training. Pretrained models from UK Biobank were applied to NAKO with a linear correction to compensate domain shift, confirming that the healthy-reference model generalises across cohorts. These clarifications are now reflected in **Section UK Biobank of the Methods** and in the newly introduced **Sections External Validation of the Methods and Results.**

"*"Healthy" subjects are used exclusively for training the model and are divided into a fixed training (80%) and validation (20%) split. The validation set is used solely for model selection and early stopping. "Unhealthy" subjects are held out entirely and used only as a test set to evaluate performance on pathological cases. To assess generalisation, we further introduce an external*

validation cohort from the NAKO study [21], which is completely unseen during training and model selection.”

[21] Bamberg, F. et al. Whole-body mr imaging in the german national cohort: rationale, design, and technical background. Radiology 277, 206–220 (2015)

8. Bias correction

The addition of a bias correction step further underscores the need for an independent healthy validation set.

Bias correction (linear correction) is a standard practice in age prediction to account for the typical underestimation of older subjects and over-estimation of yonder subjects [52]. We have added a paragraph in the Discussion to comment on this:

“*Firstly, the dataset has a limited age range with most subjects centered around 60-70 years old, which makes age prediction especially challenging for under-respresented age groups. This is a very common issue in such ageing datasets that most works address by adding a bias correction step to the pipeline as an effort to mitigate the effects of such a narrow and unbalanced age range [21].”*

However, to ensure independent validation, we have added a healthy external cohort from NAKO, which confirms the generalizability of our model introduced in **Sections External Validation of the Methods and Results.**

[21] Bamberg, F. et al. Whole-body mr imaging in the german national cohort: rationale, design, and technical background. Radiology 277, 206–220 (2015).

[52] Azzam M, Xu Z, Liu R, Li L, Meng Soh K, Challagundla KB, Wan S, Wang J. A review of artificial intelligence-based brain age estimation and its applications for related diseases. Briefings in Functional Genomics. 2025;24:elae042.

9. Hypertension and ageing

Could the authors clarify **whether hypertension is considered part of “natural ageing”**? The removal of all ICD-10 codes would exclude hypertensive cases, which are very common in UK Biobank. This exclusion could bias the training set toward an unexpectedly healthy ageing cohort.

We purposefully exclude all ICD-10 and self-reported disease codes, including hypertension, from the training cohort to model a strictly non-pathological healthy ageing process, as we are aiming to establish a baseline healthy ageing cohort. Although hypertension is prevalent in ageing populations, it is a condition associated with an increased risk of stroke and a doubling in the risk of heart disease [1], which renders it impossible to characterise as “healthy ageing”. Our results also show that hypertension is significantly associated with an accelerated age gap in the whole body, the heart and the lungs (cf Figure 4 and Supplementary Figure A2), which further highlights that it is a pathological development.

We, however, recognise that this exclusion might create an "unexpectedly healthy" cohort, and that the definition of an ideal healthy cohort is not straightforward. As such, we have added a point in the **Discussion** to address the limitations and implications of this methodological choice:

“We define our “healthy” cohort as subjects that do not have an ICD-10 record and no self-reported diseases, which might not be a sufficient characteristic to ensure their ageing is “healthy”, as some healthy subjects may harbour undetected conditions. On the contrary, this strict criterion excludes subjects with hypertension, a highly prevalent but highly treatable condition in older subjects, which

might not greatly interfere with a healthy ageing process, although its association with an increased risk of stroke and heart disease [53]. Despite its limitations, this is a commonly used approach in current studies [12].”

[12] Tian, Y. E. et al. Heterogeneous aging across multiple organ systems and prediction of chronic disease and mortality. Nature medicine 29, 1221–1231 (2023).

[53] McEniery CM, Wilkinson IB, Avolio AP. Age, hypertension and arterial function. Clinical and Experimental Pharmacology and Physiology. 2007 Jul;34(7):665-71.

10. Segmentation quality
More detail is needed on the number of faulty segmentations and how they were handled.

The Vibesegmentator [22] was trained and validated on the UKBB dataset, and demonstrated robust performances in terms of faulty segmentations. Given the detailed experiments that were performed in the original paper, we harness their work and utilise the segmentations generated by the Vibesegmentator. More details about this well-established segmentation pipeline can be found in the appendix of the paper: https://static-content.springer.com/esm/art%3A10.1007%2Fs00330-025-12035-9/MediaObjects/330_2025_12035_MOESM1_ESM.pdf

[22] Graf, R. et al. Vibesegmentator: full body mri segmentation for the nako and uk biobank. European Radiology 1–15 (2025)

Major Concern
The lack of **external validation** is the most significant limitation of this study. Without it, the robustness and clinical relevance of the findings remain uncertain.

We have added this to the paper.

REFERENCES

[8] Butler, R. N. et al. Aging: the reality: biomarkers of aging: from primitive organisms to humans. The Journals of Gerontology Series A: Biological Sciences and Medical Sciences 59, B560–B567 (2004).

[12] Tian, Y. E. et al. Heterogeneous aging across multiple organ systems and prediction of chronic disease and mortality. Nature medicine 29, 1221–1231 (2023).

[15] Bashyam VM, Erus G, Doshi J, Habes M, Nasrallah IM, Truelove-Hill M, et al. MRI signatures of brain age and disease over the lifespan based on a deep brain network and 14 468 individuals worldwide. Brain. 2020;143:2312–24.

[20] Lavdas, I. et al. Machine learning in whole-body mri: experiences and challenges from an applied study using multicentre data. Clinical radiology 74, 346–356 (2019).

[21] Bamberg, F. et al. Whole-body mr imaging in the german national cohort: rationale, design, and technical background. Radiology 277, 206–220 (2015).

[22] Graf, R. et al. Vibesegmentator: full body mri segmentation for the nako and uk biobank. European Radiology 1–15 (2025)

[23] Singh, S. P., Wang, L., Gupta, S., Goli, H., Padmanabhan, P., & Gulyás, B. (2020). 3D Deep Learning on Medical Images: A Review. Sensors, 20(18), 5097. https://doi.org/10.3390/s20185097

[24] Paszke, A. et al. Pytorch: An imperative style, high-performance deep learning library. CoRR abs/1912.01703 (2019). URL http://arxiv.org/abs/1912.01703

[25] Carreira, Joao, and Andrew Zisserman. "Quo vadis, action recognition? a new model and the kinetics dataset." proceedings of the IEEE Conference on Computer Vision and Pattern Recognition. 2017.

[28] Zhong, Z., Zheng, L., Kang, G., Li, S. & Yang, Y. Random erasing data augmentation, Vol. 34, 13001–13008 (2020).

[29] DeVries, T. & Taylor, G. W. Improved regularization of convolutional neural networks with cutout. arXiv preprint arXiv:1708.04552 (2017).

[36] Austin, P. C. An introduction to propensity score methods for reducing the effects of confounding in observational studies. Multivariate behavioral research 46, 399–424 (2011).

[52] Azzam, M. et al. A review of artificial intelligence-based brain age estimation and its applications for related diseases. Briefings in Functional Genomics 24, elae042 (2025).

[53] McEniery CM, Wilkinson IB, Avolio AP. Age, hypertension and arterial function. Clinical and Experimental Pharmacology and Physiology. 2007 Jul;34(7):665-71.

Reviewers' comments:

Reviewer #1 (Remarks to the Author):

The authors have substantially addressed all major concerns raised in the previous review. The revised manuscript is significantly improved in terms of methodological clarity, statistical robustness, and conceptual precision. In particular, the clarification of the data splitting strategy, the introduction of propensity score weighting for disease associations, the inclusion of pre- and post-bias-correction results, and the revision of the “Digital Twin” terminology represent meaningful and appropriate responses.

The study now presents a comprehensive and technically sound investigation of multi-organ ageing patterns from large-scale medical imaging data, with results that are of clear interest to the readership of the journal.

Minor comment

While the renaming of the “Digital Twin” to “Virtual Ageing Model” is appropriate and improves conceptual accuracy, the manuscript would benefit from a slightly more explicit interpretative disclaimer. The current framework relies on cross-subject organ substitution to generate counterfactual scenarios, which should be interpreted as an exploratory intervention analysis rather than a biologically causal or generative simulation of ageing trajectories within an individual. A brief clarification in the Discussion would help prevent overinterpretation by non-technical readers and further strengthen the conceptual positioning of the method.

> We thank the reviewer for this comment and have updated this passage in **Section 4 (Dicussion & Conclusion)**:
>
> *“The routine assessments of the ageing patterns of a single subject, are discussed in form of the here presented Virtual Ageing Model. We note that the VAM does not aim to provide a biological simulation of ageing but merely to explore perturbation-based counterfactual scenarios and to shed light on possible risk factors and potentially guide medical examination towards which regions show especially large age gaps.”*

Reviewer #2 (Remarks to the Author):

While the authors addressed the most of concerns raised by the reviewers, there are lingering concerns as follows:

1. What is the MAE on NAKO before and after linear correction? How do you know a linear correction is sufficient rather than masking poor generalization?

The linear correction step has been calibrated on a specific held-out set, which is not used for the subsequent analysis. This indicates that the proposed ageing model can generalise. We have clarified **Section 2** (**Methods)** to better reflect this:

"*To further validate the generalisability of the pipeline, we introduce an external validation set from the NAKO dataset. The previously described models (trained on the healthy UK Biobank data) were used to predict ages on this external validation. To account for the slight domain shift introduced by using a previously unseen dataset, we correct the estimated ages using a linear correction step (see Bias Correction Section). A held-out portion of 10% of the dataset was used to calibrate this correction for the whole body (n = 835) and the lungs (n = 765). The correction was applied to the resulting 90% of the data (n = 7522 for the whole body and n = 6893 for the lungs), which was subsequently used to assess accelerated ageing in smokers."*

To better illustrate the effect of linear correction, we have included the MAEs before and after correction as follows in **Section 3** (**Results)**:

"To assess the generalisability of the models and analyses and assess the reproducibility of our findings we introduce an external validation on the German National Cohort (NAKO) [21]. The trained models were applied on this independent dataset and a linear correction step was performed to account for systematic prediction biases (details are provided in Section External Validation). Figure 7 shows the predicted ages for smokers and non-smokers for both whole body and lungs ages on NAKO. Results were consistent with the UK Biobank analysis (Section Lifestyle and Environment), with smokers exhibiting higher age gaps than non-smokers for both whole-body and lungs. Whole-body age gaps were +0.557 years in smokers (n = 1271) versus −0.215 years in non-smokers (n = 6077), while lung age gaps were +0.727 years (n = 1177) versus −0.310 years (n = 5571), respectively. Linear correction reduced systematic prediction biases, shifting whole-body age gaps from −0.848 to −0.215 years (non-smokers) and +0.824 to +0.557 years (smokers), and lung age gaps from +3.493 to −0.310 years and +5.286 to +0.727 years respectively."

We note that the whole-body values differ slightly from the previous version because we corrected a small error in the reported sample size.

[21] Bamberg, F. et al. Whole-body mr imaging in the german national cohort: rationale, design, and technical background. Radiology 277, 206–220 (2015).

2. If SHAP is not applicable, what alternative interpretability method ensur aptures biologically meaningful signals rather than artifacts?

We thank the reviewer for this comment. We agree that justifying the interpretability approach of the Virtual Ageing Model (VAM) is important, in fact the Virtual Aging Model can be understood as an organ-level perturbation-based interpretability analysis as it directly quantifies each organ's leverage over whole-body ageing through controlled counterfactual substitution. This makes it both more tractable and more biologically meaningful than SHAP in our setting, where inputs to the whole-body predictor are latent organ-age estimates rather than interpretable tabular features. We have clarified this point in **Section 4 (Dicussion & Conclusion)**:

"The routine assessments of the ageing patterns of a single subject, are discussed in form of the here presented Virtual Ageing Model. We note that the VAM does not aim to provide a biological simulation of ageing but merely to explore perturbation-based counterfactual scenarios and to shed light on possible risk factors and potentially guide medical examination towards which regions show especially large age gaps."

Moreover, we have extended the VAM to strengthen robustness for this analysis in the revision by including more subjects and evaluating results across five seeds **(Fig 8 and Section 3 Virtual Ageing Model)**:

"In order to investigate ageing patterns of different regions in the body for individuals, we construct a "Virtual Ageing Model" – a digitised representation of a person using the MR images of the different body regions and their age predictions. This Virtual Ageing Model (VAM) allows (a) a detailed investigation of existing ageing patterns and (b) artificially changing the appearance of the person and studying the impact of local changes on the whole body on an individual level.

Figure 8A visualises an example workflow of a female subject (a random subject that shows accelerated whole-body age). For a subject that obtains MR images at a medical screening, we can create their VAM. This subject shows an overall whole-body accelerated ageing of +5.9 years. A further investigation of the body regions shows almost no accelerated ageing (age gap ≤ 1 year) in the brain, the heart, and the liver, and mildly accelerated ageing (age gap ≤ 4 years) in the lungs, the muscles, and the intestine. The most accelerated body-region of this subject is the spine with an age gap of +5.8 years. More results, including artificially changing the appearance of other body regions, are in the Appendix, Section A.5 and Table A2. To evaluate the contribution of individual organs to whole-body predicted age, we applied the VAM as a perturbation-based analysis, replacing each organ independently with an accelerated one. Figure 8B shows the resulting whole-body

predicted age (blue), alongside each subject's reference organ age (red) and the accelerated replacement organ age (green), stratified by sex. For both males and females, accelerated organ substitution consistently increased the whole-body predicted age. In females, heart substitution elevated whole-body age to 68.3 years, while muscle substitution produced the largest shift, reaching 69.7 years. Substitutions of the spine, lungs, liver, and intestine resulted in comparatively modest elevations. In males, a similar pattern emerged: heart, lungs, and intestine substitutions each elevated whole-body predicted age, with muscle again producing a notable upward effect. Across both sexes, however, the absolute increase in whole-body predicted age remained modest relative to the magnitude of the substituted organ age."

Section 2 (**Methods)** has also been updated to reflect implementation and sample size selection details:

"We follow the approach of Starck et al. [40] for whole-body MR image registration and only register subjects of the same sex to one another. For registration, a (a) reference imageis selected, to which several (b) moving images can be registered. We (a) select a random subject for each sex that does not show accelerated whole-body ageing and evaluate their region-specific age gaps. These subjects have an age of 66.1 years (female) and 64.1 (male). We then (b) select 10 subjects of the same sex that show an accelerated age for each six organ (120 in total). Figure 8B summarises the predicted whole-body and region-specific ages of the selected subjects, which are used to replace the original region of interest in the reference subject (in red). In order to ensure alignment of the individual images, we register the whole-body images of the subjects that will function as the new substituted body regions (moving images) to the selected reference image. We use Deepali [41] as the registration software and register in two steps: First, we affinely align the images and then add a deformable registration step to fine-tune the registration. We note that we cannot replace the brain images but only use the body region from the whole-body images. Subsequently, we cut out the region of interest from the respective moving image and replace the corresponding region in the reference image. We can do this for an arbitrary combination of body regions. In Figure 8, we show an example of artificially altering all accelerated body regions (spine, intestine, muscle, lungs) and compare the whole-body age predictions of the resulting prediction of the Virtual Ageing Model with the original whole-body age prediction of the reference subject over 5 seeds. Additional results can be found in the Supplementary, Section A.5."

Reviewer #4 (Remarks to the Author):

The authors have significantly improved the manuscript, and my major concerns have now been addressed.
Several key methodological choices are currently justified primarily by referencing to community norms or prior usage rather than by evidence within this study. Specifically, the manuscript would be strengthened by providing additional empirical justification for:
• the choice of a single, uniform architecture across all organs,

> Regarding the choice of a single, uniform architecture across all organs: our primary goal is to demonstrate the feasibility of a unified framework for analysing region-specific ageing patterns, rather than to optimise individual organ predictors. Using a single, uniform architecture ensures comparability across regions and avoids confounding effects. We agree that investigating organ-specific architectures could potentially improve performance but is beyond the scope of this study and would require extensive additional compute resources. We have clarified this point in the manuscript and note it as a direction for future work as follows in **Section 4 (Dicussion & Conclusion)**:
>
> "*While our work renders interesting results about global and local ageing patterns in medical images, we note some limitations. Firstly, the dataset has a limited age range with most subjects centered around 60-70 years old, which makes age prediction especially challenging for under-respresented age groups. This is a very common issue in such ageing datasets that most works address by adding a bias correction step to the pipeline as an effort to mitigate the effects of such a narrow and unbalanced age range [52]. However, an investigation of a dataset with a wider age range would be highly interesting. We furthermore employ a single, uniform network architecture across all organs to ensure comparability between regions. Our goal is to demonstrate the feasibility of a unified framework for analysing region-specific ageing patterns rather than to optimise individual organ predictors. Investigating organ-specific architectures could further improve performance and is an interesting direction for future work.*"
>
> [52] Azzam, M. et al. A review of artificial intelligence-based brain age estimation and its applications for related diseases. Briefings in Functional Genomics 24, elae042 (2025).

• the use of the masking strategy during training, and

> We thank the reviewer for this comment. We have added this ablation and now provide direct empirical evidence within the study. Figure A1 compares the residual age-gap distributions of the masked and unmasked models for the training and validation splits across all seeds. The masked model shows a better match between the training and validation distributions, suggesting slightly better robustness to overfitting. This demonstrates that masking acts as an effective regulariser, reducing the model's reliance on spurious training correlations and improving generalisation to unseen

subjects. We have added **Supplementary Figure A1** displaying this in the **Supplementary Material A** and refer to it as follows:

"In order to prevent the model from focusing on only highly predictive regions in the body, such as the spine and the cardiac area, we randomly mask out parts of the images during training to force the model to use the whole available information to predict a subject's age. We use this "masking" as a regularisation technique [28, 29] for the whole-body and the brain age predictors, an empirical justification for this training strategy can be found in the Supplementary Material, Figure A1. Furthermore, we use random data augmentation (addition of noise, rotation) applied exclusively to the training set during training for all models to prevent over-fitting. For the region-specific deep learning models, we do not apply any masking, since the areas of interest are already limited to small parts of the whole image and we expect the field of view to be large enough to prevent the model from focusing on highly specific sub-regions."

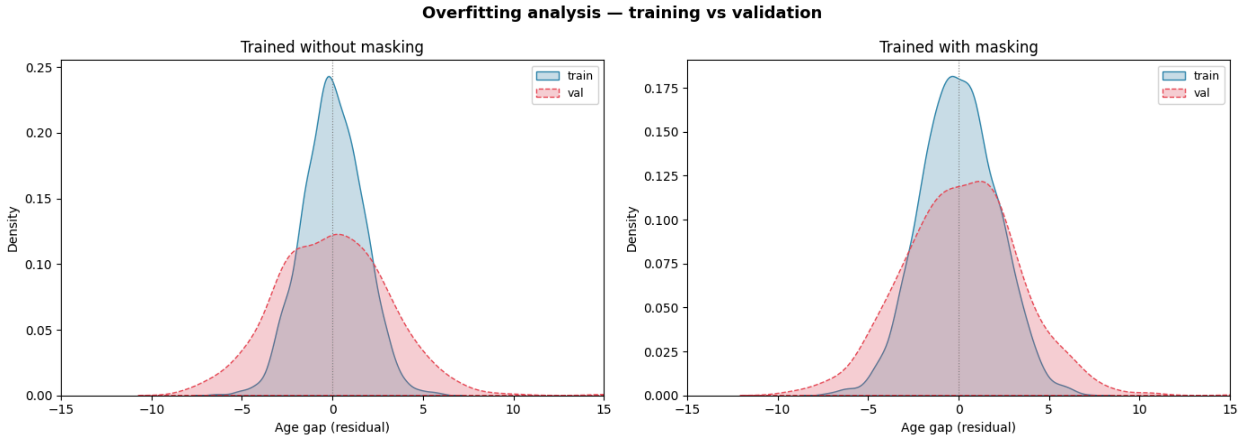

• the application of the bias correction step.

We have added a visualisation showing the fitted relationship between the raw age gap and chronological age before and after correction in **Supplementary Figure A3**. This illustrates the reduction of the regression-to-the-mean bias in the best cases and shows no noticeable effect otherwise. This behaviour is also reflected in the reported mean absolute errors (MAE) in **Supplementary Table A1**, before and after applying the correction. We have furthermore added a paragraph **Impact of the Bias correction** to the manuscript:

"*Bias correction is performed to mitigate the regression-toward-the-mean effect of ageing datasets. To assess the impact of this step, we analysed both the fitted relationship between the raw age gap and chronological age as well as the resulting prediction errors before and after correction. Supplementary Figure A.2 visualises the fitted linear*

relationship used for bias correction and illustrates the effect of the correction on the regression-to-the-mean bias. In several cases, the correction reduces the age-dependent bias visible in the raw age gap, while in other cases the relationship is weak and the correction has little observable effect. The quantitative impact of the correction is summarised in Supplementary Table A1, which reports the mean absolute error (MAE) for all models on the training, validation, and test sets before and after applying the bias correction. These results indicate that the correction successfully mitigates age-dependent bias where present without negatively affecting model performance when such bias is minimal."

While these choices are reasonable and commonly used, the current justification largely rests on "others have done this before." The authors could consider sensitivity analyses, ablations, or supplementary comparisons to strengthen/increase confidence in the results.

Reviewers' comments:
Reviewer #2 (Remarks to the Author):

I thank the authors for their careful revision. The authors have addressed prior critiques adequately, and I have no further concerns.

We thank all reviewers for their time and valuable input.
